# Fluorescent Nanocomposite of Embedded Ceria Nanoparticles in Crosslinked PVA Electrospun Nanofibers

**DOI:** 10.3390/nano6060102

**Published:** 2016-06-01

**Authors:** Nader Shehata, Soha Gaballah, Effat Samir, Aya Hamed, Marwa Saad

**Affiliations:** 1 Department of Engineering Mathematics and Physics, Faculty of Engineering, Alexandria University, Alexandria 21544, Egypt; aya_ali@mena.vt.edu; 2 Center of Smart Nanotechnology and Photonics (CSNP), SmartCI Research Center, Alexandria University, Alexandria 21544, Egypt; soha_gaballah@mena.vt.edu (S.G.); effat_samir@mena.vt.edu (E.S.); marwahamedo@gmail.com (M.S.); 3 The Utah Science Technology and Research Agency (USTAR) Bioinnovations center, Utah State University, Logan, UT 84341, USA; 4 Department of Chemical Engineering, Faculty of Engineering, Alexandria University, Alexandria 21544, Egypt; 5 Department of Electrical Engineering, Faculty of Engineering, Alexandria University, Alexandria 21544, Egypt

**Keywords:** ceria nanoparticles, electrospinning, nanofibers, crosslinking, fluorescence

## Abstract

This paper introduces a new fluorescent nanocomposite of electrospun biodegradable nanofibers embedded with optical nanoparticles. In detail, this work introduces the fluorescence properties of PVA nanofibers generated by the electrospinning technique with embedded cerium oxide (ceria) nanoparticles. Under near-ultra violet excitation, the synthesized nanocomposite generates a visible fluorescent emission at 520 nm, varying its intensity peak according to the concentration of *in situ* embedded ceria nanoparticles. This is due to the fact that the embedded ceria nanoparticles have optical tri-valiant cerium ions, associated with formed oxygen vacancies, with a direct allowed bandgap around 3.5 eV. In addition, the impact of chemical crosslinking of the PVA on the fluorescence emission is studied in both cases of adding ceria nanoparticles *in situ* or of a post-synthesis addition via a spin-coating mechanism. Other optical and structural characteristics such as absorbance dispersion, direct bandgap, FTIR spectroscopy, and SEM analysis are presented. The synthesized optical nanocomposite could be helpful in different applications such as environmental monitoring and bioimaging.

## 1. Introduction

Cerium oxide nanoparticles (ceria NPs) attract great research and commercial interests due to their observable capability to capture radicals and dissolved oxygen [1,2]. These promising properties are applicable in many applications related to the medical, environmental, and sustainable energy fields [3,4,5]. Depending on the oxidation state of cerium ions, ceria could exist in two forms: active with associated charged oxygen vacancies (Ce^3+^) and non-active with no formed O-vacancies (Ce^4+^) [6]. The formed O-vacancies act as probes to scavenge some charged objects such as radicals and dissolved oxygen. The conversion of solid oxide from a lower oxidation state (Ce^3+^) to a higher one (Ce^4+^) occurs in a fast manner while the opposite reaction is difficult to achieve [7]. Therefore, it is a challenge to keep ceria NPs in active form, containing Ce^3+^ ions to be fitted into the previously mentioned applications. Ceria nanoparticles can be in the form of either dry powder or colloidal particles in solution. However, there is a lack of research in forming the active ceria nanoparticles in a solid host.

In this paper, we have produced active ceria NPs in a nanofiber host. In a research work done by Yang *et al.*, it was showed that the ceria formed on nanofibers has a non-active form (Ce^4+^; CeO_2_) [8]. However, our work suggests the surface decoration of electrospun nanofibers made from poly (vinyl alcohol) (PVA) as host media with the active ceria NPs. PVA polymer is considered one of the well-known polymers as it is a non-toxic, cheap, and biodegradable water-soluble polymer [9]. Regarding the synthesis technique of the nanoparticles, ceria nanoparticles are prepared using the chemical precipitation technique due to its simplicity and cheap chemical precursors [10]. Then, the embedded ceria nanoparticles with PVA solution are mixed together as the solution feeder in the electrospinning process to generate the final nanofibers. The electrospinning technique is preferred due to its simplicity of operation, the feasibility to embed ceria nanoparticles in the resulting PVA nanofibers, and the potential for scale-up to manufacture large volumes [11,12]. In this process, the formed high electric field between both the needle and target affects on the polymer droplet at the needle tip to stretch and to deposit into fibers at the surface of the target [13]. The resulting webs can be feasible for many applications such as environmental monitoring, tissue and biomedical engineering, filtration media, fuel cells, electronics, and optical devices [14,15,16]. Compared to other fluorescent electrospun nanofibers in other literature reviews such as in Reference [14], the fluorescence of the electrospun nanofibers depends on the produced fibers’ morphology, which results in challenging electrospinning processes to determine the optimal conditions, while the introduced electrospun PVA solution embedded with ceria nanoparticles depends only on the concentration of ceria nanoparticles in the produced fibers with the same PVA electrospinning conditions. As another contribution, to solve the problem of the formed hydrophilic nanofibers of PVA which can be easily dissolved in any water-based solution, we have used the chemical esterification method using malic acid. Hence, the produced nanofibers can resist solubility and become a hydrophobic material, which could be helpful in further applications including sensors and biomedical materials. Another novel method is introducing ceria NPs in the crosslinked PVA nanofibers whether *in situ* or in post-synthesis treatment using the spin-coating method on crosslinked electrospun PVA nanofibers (NFs). This method shows promising results in optical sensor applications such as peroxide [17].

## 2. Results

In this presented work, two different methods are introduced to produce fluorescent electrospun nanofibers, which are electrospun PVA nanofibers created with the embedded *in situ* ceria nanoparticles technique and the deposited ceria nanoparticles within a crosslinked PVA nanofiber technique. The embedded *in situ* ceria nanoparticle concentration can be controlled in PVA electrospun nanofibers with less ceria agglomeration inside the fiber [18].

The new contribution is depositing ceria nanoparticles in a crosslinked electrospun PVA nanofiber surface. However, this technique shows some difficulty in controlling the ceria nanoparticle concentration due to the losses that happen during the deposition technique; also ceria nanoparticle agglomerations were difficult to control, leading the presence of florescence quenching mechanism compared to the first technique, though it can be used in some applications such as drug delivery that deal with the crosslinked nanofibers mat as ceria nanoparticle carriers or a cohesive media. Despite the used deposition technique, ceria nanoparticles show that they are still active, as discussed in the next section.

### 2.1. Optical Characterization of Ceria NPs Embedded inSitu with PVA Nanofibers

**Figure 1:**
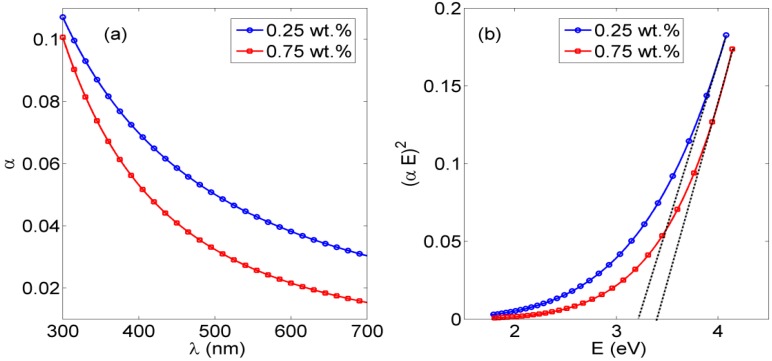
Poly(vinyl alcohol) (PVA) nanofibers (NFs) with *in situ* embedded cerium oxide nanoparticles (ceria NPs):(**a**) Absorbance curve;(**b**) Bandgap curve.

The absorbance curves of PVA NFs embedded *in situ* in ceria NPs with concentrations of 0.25% and 0.75% are shown in Figure 1a. This absorbance raise to 400 nm is correlated to the embedded ceria NPs, as the PVA nanofibers alone, whether crosslinked or not, show no absorbance at all over the detected range of wavelengths based on experimental testing. The optical allowed direct bandgap can be calculated directly from the obtained absorbance curves using the following Equation (1) [19].

(1) $$\alpha (E)=A{\left(E-E_{g}\right)}^{1/2}$$

where α is the absorbance coefficient, *A* is a constant that depends on the effective masses of electrons and holes in ceria NPs, *E* is the absorbed photon energy, and *E_g_* is the allowed direct bandgap. Figure 1b shows the relation between (α*E*)^2^ *versus E*, and the intersection of the extrapolation of the linear part of the (α*E*)^2^ curve with the *E* (*x*-axis) is equal to the allowed direct bandgap *E_g_* is the particular composition of ceria NPs.

**Figure 2:**
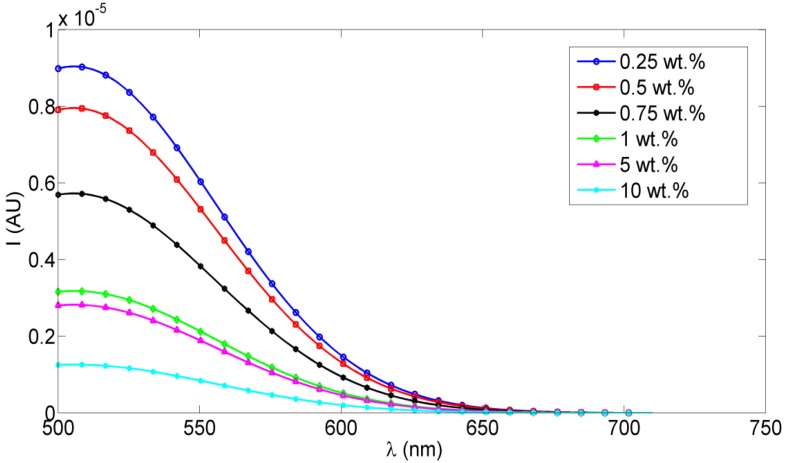
Fluorescence intensity PVA NFs with *in situ* embedded different concentrations of ceria NPs.

**Figure 3:**
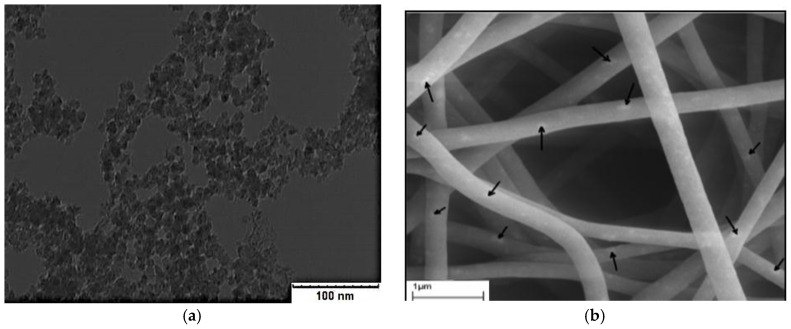
(**a**) TEM of ceria NPs and (**b**) SEM of nanofibers with embedded ceria with the arrows showing the agglomerated parts of ceria NPs over the PVA nanofibers.

Fluorescence intensity measurements were obtained for different concentrations of ceria NPs which were embedded *in situ* in PVA NFs. Studied concentrations of ceria NPs as examples were <1 wt %, 5 wt %, and 10 wt %. As shown in Figure 2, the fluorescence emission appears at a wavelength approximately equal to 520 nm under 430 nm excitation, which is one of the embedded ceria NPs’ optical characteristics [20], with no fluorescence peak emitted from the non-optical mat of PVA-only nanofibers based on our experimental measurements. The TEM image of ceria nanoparticles and the SEM image of PVA nanofibers with embedded ceria nanoparticles *in situ* are shown in Figure 3a,b. The average grain size of ceria nanoparticles is ~6 nm and the formed nanofibers’ mean diameter is ~200 nm. From Figure 3b, it can be shown that some ceria nanoparticles are agglomerated on the electrospun nanofibers.

### 2.2. Effect of Crosslinking with Ceria NPs “in Situ” or Spin-Coated

**Figure 4:**
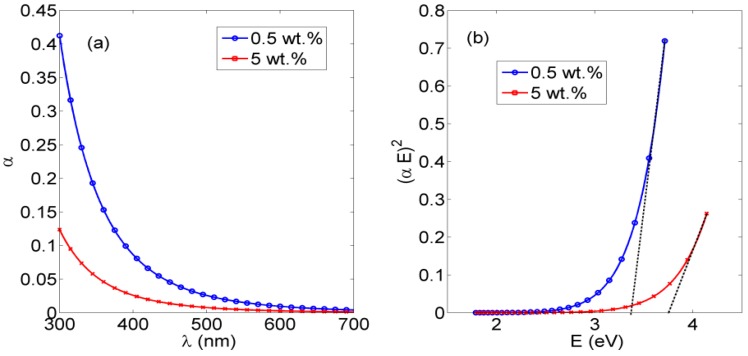
(**a**) The absorbance dispersion of crosslinked PVA NFs with *in situ* embedded different concentrations of ceria NPs, and (**b**) the corresponding direct allowed bandgap.

**Figure 5:**
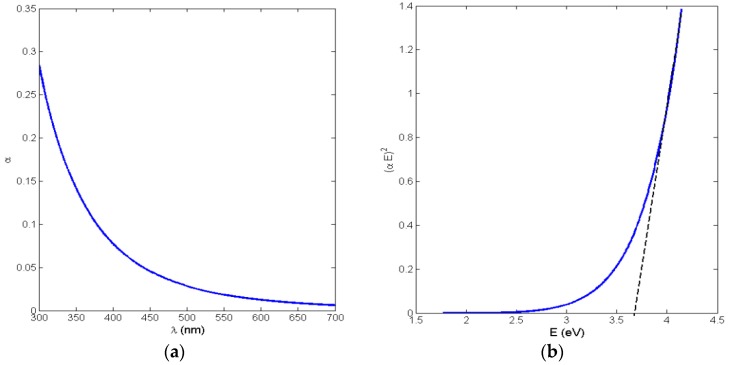
(**a**) The absorbance dispersion of spin-coated ceria NPs at 1 wt % on crosslinked PVA NFs, and (**b**) the corresponding allowed direct bandgap.

**Figure 6:**
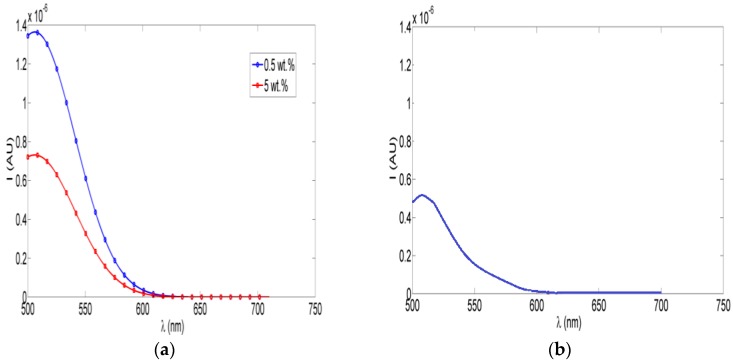
(**a**) Fluorescence intensity of crosslinked PVA NFs with *in situ* embedded different concentrations of ceria NPs; (**b**) Fluorescence intensity after immersing the crosslinked fiber in water.

**Figure 7:**
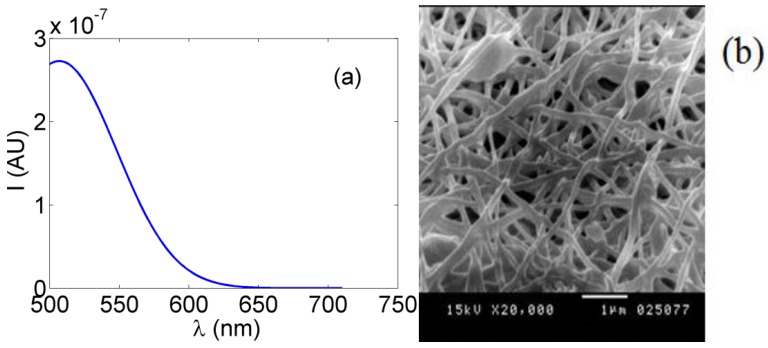
(**a**) Fluorescence intensity of spin-coated ceria NPs on crosslinked PVA NFs and (**b**) SEM image of the crosslinked PVA NFs.

As discussed before, the esterification method is used as a chemical crosslinking method to convert the generated NFs from being a soluble mat to being a hydrophobic one that can resist dissolving in water. Here, the optical characterizations of crosslinked PVA NFs with *in situ* embedded ceria NPs at concentrations of 1 wt % and 5 wt % are studied, as are ceria NPs spin-coated on the mat of crosslinked PVA NFs as a post-addition step of the nanoparticles over the nanofibers, as shown in both Figure 4 and Figure 5. Both figures show the absorbance and bandgap for *in situ* embedded ceria NPs and spin-coated ceria NPs on a mat of crosslinked PVA NFs, respectively. Regarding fluorescence intensity, as shown in Figure 6a and Figure 7a, the fluorescence intensities of crosslinked PVA NFs embedded with *in situ* ceria NPs at 1 wt % and ceria NPs spin-coated on crosslinked PVA NFs have the same behavior as discussed in non-esterified NFs. That gives a promising conclusion that our electrospun nanocomposite can be optically fluorescent in addition to being hydrophobic. Figure 6b shows the reduced fluorescence intensity.

**Figure 8:**
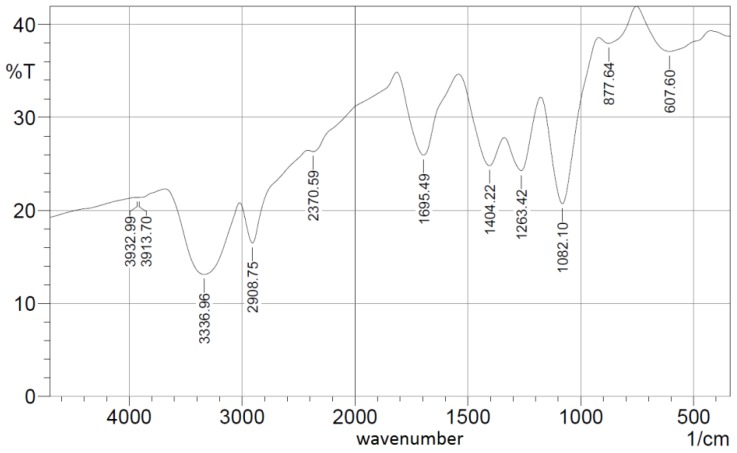
FTIR spectroscopy pattern of crosslinked PVA with embedded ceria NPs.

Figure 7b presents the SEM image of the chemically crosslinked PVA nanofibers due to esterification and Figure 8 shows the FTIR spectrum of the crosslinked synthesized nanocomposite.

## 3. Discussion

From Figure 1b, the resulting allowed bandgap range matches other literature which confirms the formation of active ceria with some quantity of tri-valiant cerium ions with corresponding O-vacancies [10,21]. From the fluorescence graphs in Figure 2, in the range of <1 wt % of ceria NPs, the fluorescence intensity peak increases with increasing the concentration due to more formed optical tri-valiant states of cerium ions with corresponding electron transitions of 5d–4f levels [22]. However, the increase of a higher ceria NP concentration above 1 wt % leads to a decrease in the fluorescence emission intensity peaks. This may be due to a static fluorescence quenching effect which can dominantly appear at higher ceria NP concentrations greater than 1 wt %, leading to this decrease in the fluorescence emission. From these experimental results, we can conclude that optimal concentrations of ceria NPs embedded *in situ* in PVA NFs that give a higher fluorescence intensity are less than 1 wt %.

As the optically effective materials are ceria NPs, the absorbance and bandgap curves in both Figure 4 and Figure 5 are slightly different in each method; however, all the resulting bandgap values are in the accepted ranges of active ceria NPs, around 3.5 eV, which was shown before for non-crosslinked nanofibers with embedded ceria. All of these experimental results indicate that optical properties of ceria NPs are not affected much by the crosslinking technique. However, it could be estimated that ceria nanoparticles are more optically active within the *in situ* embedding technique than in the spin-coating one, due to the smaller obtained direct allowed bandgap and the higher value of the fluorescence intensity peak.

Figure 7b shows the reduction in the average nanofibers’ mean diameter, compared to the non-esterified case, in range of 150 nm but with less porosity and a higher possibility of beads, compared to the non-esterified PVA electrospun nanofibers as shown earlier in Figure 5b. In FTIR spectroscopy as shown in Figure 8, most of the original peaks of both PVA and malic acid are not affected by adding ceria NPs. That gives evidence that the polymeric host of ceria keeps its original chemical bonds such as OH alcohol, free hydroxyl, C–H alkane, C–O carboxylic acid, ester and C=O carboxylic bonds.

## 4. Materials and Methods

### 4.1. Chemicals

All chemicals are used as received without further purification. Cerium (III) chloride heptahydrate (99.9%), Mowiol 10-98 Poly(vinyl alcohol), Malic acid are purchased from Sigma-Aldrich (St. Louis, MO, USA). Methanol, ethanol and hydrochloric acid were purchased from an Egyptian local chemical agent (Alexandria, Egypt) as commercial grade solutions.

### 4.2. Nanoparticles Synthesis

Undoped ceria nanoparticles are prepared using chemical precipitation technique similar to Chen *et al.*, but with some modifications [23]. Cerium (III) chloride heptahydrate of 0.5 g is inserted into a beaker with adding 40 mL of distilled water, and the solution is stirred using a magnetic stirrer at rate of 500 rpm for 24 synthesis process. The solution is heated to 50 °C in a hot water bath for two hours with added 1.6 mL of ammonium hydroxide. Then, it is stirred for 22 h at room temperature. The long period of stirring fractures any remaining nanorods into nanoparticles. The solution is then centrifuged, washed with de-ionized water and ethyl alcohol to remove any unreacted cerium chloride and ammonia.

### 4.3. Polymers Preparation with Embedded Nanoparticles

A concentration of 10 wt % PVA solution is prepared by mixing 10 g PVA pellets with distilled water of 90 mL. The solution is heated to 100 °C for 30 min then it was stirred overnight. This paper introduces different methods of producing PVA nanofibers with embedded ceria NPs. First, ceria NPs with different weight percentages of (0.25%, 0.5%, 0.75%, 1%, 5%) are added *in situ* to the PVA solution. The mixture is stirred for 30 min before it is entering the electrospinning process. Further, it would be shown another way to add ceria as post-synthesis treatment by spin-coating on the electrospun nanofibers.

### 4.4. Electrospinning Process

**Figure 9:**
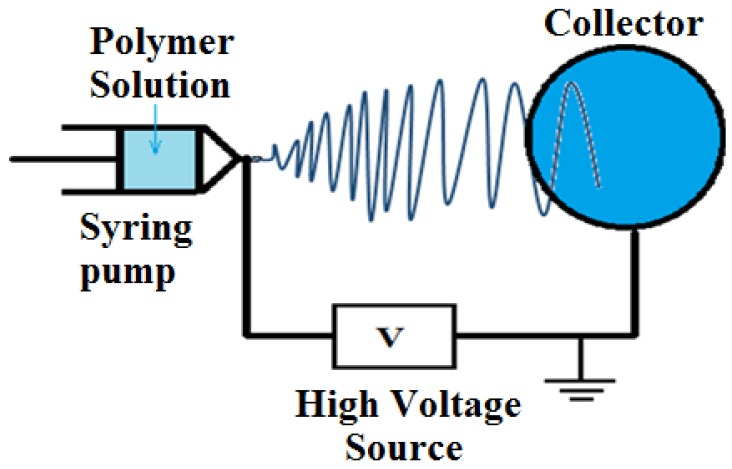
Schematic diagram of the electrospinning setup.

The electrospinning setup consists of high voltage power supply (Spellman High Voltage Electronics corporation model CZE1000R (Hauppauge, New York, NY, USA), a syringe pump (NE1000-Single Syringe Pump, (New Era, Farmingdale, New York, NY, USA) which is used to regulate the pumping rate of polymer solution, a 5 mL plastic syringe with 18 gauge metallic needle to store the polymer solution, and a circular metallic collector of radius 10 cm covered with aluminum foil is used as a target. A schematic of electrospinning setup is shown in Figure 9. The voltage power supply is connected to the needle while the collector is grounded. The distance between the needle tip and the collector is fixed at 15 cm. The voltage difference between the needle and target is 25 kV, with a flow rate of the polymer solution at 2 mL/h for 30 min running time per sample.

### 4.5. Crosslinking Procedure

Vapor phase esterification process was done in oven on two subsequent steps similar to the procedure mentioned in Reference [24], but with some changes. In the first step, electrospun nanofibers of PVA only or PVA with embedded ceria were placed in a container along with a small amount of malic acid (1–2 g) with added few droplets of HCl. The container was sealed from the ambient moisture and placed in an oven at 80 °C for 15 min. Esterification was produced via heterogeneous reaction during the first stage of 15 min. In the second step, the sample was cured for 20 min in an oven at 120 °C. Through the discussed esterification technique, the produced NFs become crosslinked and more resistive to solubility as a hydrophobic material. Then, we have ceria nanoparticles hosted by electrospun crosslinked PVA nanofibers in two forms. Firstly, ceria NPs were added *in situ* with the PVA solution before being electrospun and esterified. Secondly, ceria nanoparticles were added as a post-synthesis step on the crosslinked electrospun PVA-only nanofibers through spin-coater, MTI-100 model (Richmond, CA, USA), at a speed of 500 rpm.

### 4.6. Characterization of the Synthesized Nanocomposite

**Figure 10:**
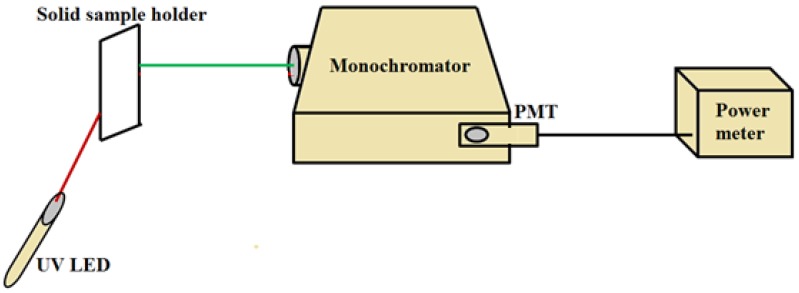
Fluorescence intensity spectroscopy setup.

PVA nanofibers with embedded ceria NPs, whether *in situ* added or spin-coated, are optically characterized by measuring its optical absorbance, and fluorescence intensity curves. Optical absorbance in a wavelength within range from 300 to 700 nm was measured by using ultra violet-visible (UV-Vis) spectrophotometer of model (PG T92+) (Beijing, China). From absorbance curves, corresponding band gap of the formed nanocomposite can be determined, as will be discussed in next section. Fluorescence intensity measurements have been detected by a hand-made fluorescence spectroscopy setup, as shown in Figure 10. The used fluorescence setup is composed of UV light emitting diode (LED, Thorlab) (Newton, NJ, USA) with 430 nm excitation wavelength, Monochromator (Newport cornerstone 130) (Newport, Irvine, CA, USA) which was set to obtain fluorescence intensity at wavelength from 500 to 700 nm, Oriel photomultiplier tube (PMT) (Newport PMT77340) (Newport, Irvine, CA, USA) as a fluorescence intensity detector, and a power meter (Newport 1918-R) (Newport, Irvine, CA, USA) to display PMT detection readings. Fluorescence intensity was measured by positioning NFs solid sample holder inclined by 45° between UV-LED and the input port of the monochromator, so that the input optical signal to the monochromator is perpendicular to the initial LED excitation signal for minimum scattering effect [10]. The output port of the monochromator was directly connected with PMT, which was directly connected to 1918-R power meter.

The mean synthesized nanoparticle size was observed by transmission electron microscopy (TEM, JEOL) (Tokyo, Japan), with accelerating potential of 80 kV. Surface morphology and diameter size of electrospun nanofibers before and after esterification were investigated using scanning electron microscopy (FEI Quanta 200) (Hillsboro, OR, USA). After sputter-coating with gold, the fiber size distribution of randomly selected SEM micrograph was measured using Image-J software. The formed nanocomposites have been characterized using FTIR spectroscopy (Varian 670-IR) (Santa Clara, CA, USA).

## 5. Conclusions

This paper presents the study of adding the property of optical fluorescence to electrospun PVA nanofibers through embedding ceria nanoparticles using different techniques. In this work, ceria nanoparticles with a greater Ce^3+^ ionization state have been added *in situ* to a PVA solution and then generated together as nanofibers via the electrospinning technique. In addition, to make the electrospun fiber hydrophobic mat, PVA nanofibers have been esterified using malic acid. In this case, ceria nanoparticles are added, whether *in situ* or on the esterified nanofibers as a post-synthesis treatment using spin-coating. In all synthesized nanofibers, the active ceria nanoparticles still keep their direct bandgap value around 3.5 V, which indicates that it is still optically active. All the synthesized nanofibers are shown to be visibly fluorescent under 430 nm optical excitation. The crosslinked nanofibers using esterification show a smaller mean diameter with greater number of beads compared to the non-esterified fibers. This new optical biodegradable nanofiber mat could be applicable in a wide variety of applications, including environmental sensors and cancer treatment.

## Abbreviations

The following abbreviations are used in this manuscript:

Ceria NPs	Cerium oxide nanoparticles
PVA	Poly(vinyl alcohol)
TEM	Transmission Electron Microscope
SEM	Scanning Electron Microscope
FTIR	Fourier Transform Infra-red Spectroscopy
NFs	Nanofibers
